# The Axial Age and the Problems of the Twentieth Century: Du Bois, Jaspers, and Universal History

**DOI:** 10.1007/s12108-015-9254-0

**Published:** 2015-03-17

**Authors:** John D. Boy

**Affiliations:** Department of Sociology, University of Amsterdam, Amsterdam, The Netherlands

**Keywords:** Axial age, Universal history, Hegel, Civilizations, Temporality

## Abstract

The axial age debate has put big questions of social and cultural change back on the agenda of sociology. This paper takes this development as an occasion to reflect on how social thought works with (and against) nineteenth-century intellectual traditions in its efforts to understand history on a macro scale. Karl Jaspers, who initially formulated the axial age thesis in *The Origin and Goal of History*, revised the Hegelian account of world history by broadening the scope of the narrative to encompass all civilizations participating in the events of the first millennium BCE that saw the rise of major philosophical and religious traditions. However, his account, like the earlier philosophical accounts he seeks to improve upon, privileges cognitive developments over material practices and social interactions, and as such offers little to those seeking to make sense of how cultural patterns interact with others and spread. Here another social theorist engaging with Hegel, W. E. B. Du Bois, provides a helpful contrast. His account of the development of double-consciousness in “Of Our Spiritual Strivings,” the opening chapter of *The Souls of Black Folk*, helps us to understand experiences of encounter and the perduring historical effects they may have. Du Bois’ relational theory reminds us of the importance of unpacking abstractions and understanding processes in terms of social interactions.

The recent high-profile discussion around the “axial age”—the approximately 500-year period in the first millennium BCE in which many of the world’s religious and philosophical traditions have their origin—has once again put what Max Weber referred to as “problems of universal history” on the agenda of sociology. Long after the works of scholars like Pitrim Sorokin, Arnold Toynbee and Oswald Spengler ceased inspiring scholars to pursue macrohistorical inquiry, sociology’s recent civilizational turn opens up the prospect that the discipline may once again produce what the anthropologist (Graeber 2011) calls “big” books—those “asking big questions, meant to be read widely and spark public debate”—rather than highly specialized, piecemeal studies. Such work, however, is inevitably produced in the shadow of the nineteenth century and its intellectual traditions, from whence a set of assumptions about progress, development and modernization have been passed down that are justifiably considered problematic. This is among the reasons why “grand narratives” were viewed with suspicion in sociology for the past several decades.

This paper is a reflection on the history of social thought and on how it has worked with and against the nineteenth-century tradition of thinking about big questions, specifically the Hegelian account of world history. By turning to social thinkers of the twentieth century who sought to revise this intellectual tradition in order to think about the problems they were faced with at the time, I seek to shed light on how we might address big questions in the twenty-first century.

As is well known by now, before it initially entered the vocabulary of sociology in the 1960s, the notion of the axial age was initially formulated by the philosopher Karl Jaspers (see Wittrock 2005; Joas 2012; Boy and Torpey 2013).^1^ Written in the aftermath of the Second World War which Jaspers had spent in “internal emigration,” his work on the philosophy of history, *The Origin and Goal of History* (Jaspers 1953), tried to salvage the “spirit of Europe” in the midst of postwar devastation.^2^ In writing *Origin and Goal*, Jaspers drew on previous work in the philosophy of history, and he acknowledges the influence of G. W. F. Hegel’s conception of world history in particular. Jaspers’s project can be read as an effort to rid the Hegelian conception of world history of its overly Christo-centric framework so it could contain a multiplicity of historical experiences. He wanted to move beyond the particularity of Hegel’s Eurocentric system to a more encompassing, universal conception. One way to read Jaspers’s world history, then, is as an *Aufhebung* of Hegelian world history. *Aufhebung* (usually translated as “sublation”) is a dialectical term found in the work of Hegel that means both “to cancel out” and “to preserve.” Sublation is Hegel’s strategy to overcome one-sided positions, such as empiricism, while preserving their truth content. As I elaborate further below, Jaspers’s sublation of Hegelian world history, his attempt at overcoming its one-sidedness, hinges on the concept of the axial age.

In order to further illuminate and evaluate the way Jaspers sought to step out of the shadow of the nineteenth century in his conception of history, I will compare Jaspers’s intellectual project with another that, although conceived a half-century earlier and on the other side of the Atlantic, bears enough formal similarities to make the comparison worthwhile. W. E. B. Du Bois enrolled at Fisk College in Tennessee in 1885, less than a decade after the United States gave up on post-Civil War Reconstruction. While originally from the North, he moved to the Jim Crow South at the outset of his academic career. Between the early stages of his studies and the turn of the century, Du Bois developed an acute awareness for the reality of race and the problem of what it meant to be black in the United States during a time of neoslavery, vigilante violence, and legislated segregation. The first culmination of his consideration of this problematic was the publication, in 1903, of *The Souls of Black Folk*, a book of essays begun about five years prior to its publication (Du Bois 2007). It is a very different kind of book than Jaspers’s *Origin and Goal*, but it serves as a useful comparison because it, too, seeks to develop and account of world history in dialog with Hegel. To be clear, I am not trying to argue that Du Bois’s masterful work is derivative of Hegel, any more than Jaspers’s is merely derivative. But I follow Shamoon Zamir (1995), Paul Gilroy (1993), and Robert Gooding-Williams (1987), among others, in recognizing Hegel’s philosophy as an important aspect that shaped Du Bois’s thinking. By recognizing this affinity, it becomes possible to envision and recognize Du Bois as part of the classical tradition of social thought from which he often remains excluded (Rabaka 2010).

## The Axial Age and the Spirit of Europe

Prior to his internal emigration during the years of Nazi rule, Karl Jaspers was a professor of psychiatry and philosophy at the university in Heidelberg, Germany’s oldest (for further biographical detail, see Kirkbright 2004). Heidelberg between the world wars provided an intellectual environment in which some of the big books that influence our thinking to this day were produced by a loose group of towering scholarly figures that included, until his departure for Vienna and Munich in 1919, Max Weber, as well as his younger brother Alfred Weber, Karl Mannheim, and Norbert Elias (Blomert 1999; Remy 2002; Green 1974). Other young intellectuals in the Heidelberg circle during this time who later rose to prominence include Marxist philosophers Ernst Bloch and György Lukács, as well as Alexandre Kojève, a Hegel scholar who would later teach in Paris. Given this impressive array of intellectual talent, the characterization by an immediate postwar observer of Heidelberg as an “exterritorial enclave of world-spirit” is not entirely off the mark (Sombart 2000). As such, the Heidelberg milieu constitutes a kind of bridge between nineteenth-century and twentieth-century social thought. George Steinmetz (2010) has argued, too, that Weimar-era Heidelberg scholars gave rise to a distinct school of thinking about history that has left a lasting mark on sociology. It is important to understand the basic assumptions Jaspers made about history when formulating the axial age thesis, and this is the time and place in which they were formed.

Jaspers and Max Weber became acquainted before the First World War, and the influence of the great sociologist on the philosopher is apparent. Weber died in 1920 at the relatively young age of 56, however, so in the three decades before the publication of *Origin and Goal*, Jaspers’s thinking developed in conversation with other scholars in his social circle. It would be fallacious to make too much of Jaspers’ connection to Max Weber or to think of him simply as a “Weberian.” Throughout the interwar years, Jaspers often joined his colleagues at the Institute for Social and Policy Studies (Insosta) for talks on sociological topics. He also participated in the Sunday afternoon salons held at the house of Marianne Weber, Max Weber’s widow (M. Weber 1977). Additionally, Jaspers and Alfred Weber were in close communication for many years. The two shared a number of students, and they continued to work closely together after the war. One of the most famous members of the first generation of sociologists, Norbert Elias, a student of Alfred Weber’s from 1925 to 1930, initially came to Heidelberg to study philosophy under Jaspers.

Elias’s famous work on “the civilizing process,” first published in 1939, begins with an analysis of the obsession among German intellectuals with the antithesis between soulful Germanic *Kultur* and coldly calculating *Zivilisation* (Elias 1978). While at the time Elias’s point of reference was Thomas Mann’s *Reflections of a Nonpolitical Man*, he did not have to look to the northern German novelist for an example of an intellectual who insisted on the distinction between culture and civilization. His elder colleagues in Heidelberg, including Alfred Weber and Jaspers, also insisted upon this division, for instance in their debate on relativism with Karl Mannheim, whose *Ideology and Utopia*, a foundational text in the sociology of knowledge, suggested a “relativization of spirit” that Jaspers found intolerable. He condemned the book harshly in his 1932 collection, *Die geistige Situation der Zeit* (Jaspers 1933, 149–51). Blomert (1999, 213) accounts for Jaspers’s strong rejection of Mannheim’s standpoint as follows:

Mannheim’s relationism affected them in the depths of their understanding of self and culture in ways that they could not parry. In their helplessness, they could only grasp it as nihilism. The realm of spirit, the Goethean understanding of humanity, was so self-evident to them, so religiously internalized, that they readily recognized the assault implied by Mannheim’s thought, but found themselves unwilling and unable to grapple with this new manner of thinking.

This belief stuck with Jaspers into the postwar years when he wrote *Origin and Goal*. He continued to believe that an account of the history of humanity as a whole had to be about what he took to be the essence of what it means to be human, namely the realm of spirit.

The introduction to *Origin and Goal* describes the paradoxical position the book seeks to argue. On the one hand, Jaspers rejects philosophy of history in the then conventional sense—the genealogy of which Jaspers traces, much like Alfred Weber (1935), “from Augustine to Hegel”—but he also does not believe strictly empirical historiography to be sufficient. If history is to be existentially meaningful and not just an aggregation of facts, it must make distinctions between more or less meaningful data. For this purpose Jaspers requires some means by which to differentiate between that which has universal significance, and that which has merely local significance. This need to marry his historical thought with a logical system takes Jaspers to the work of Hegel. In fact, no other thinker is as prominent in Jaspers’s treatment of world history as the Swabian philosopher. Jaspers cites Hegel more than some of the more immediate sources of the concept of the axial age, such as Alfred and Max Weber or, to go back even further, Ernst von Lasaulx and Viktor von Strauß. In fact, no person listed in the index of the English translation has as many entries as Hegel, who appears in all three parts of his book and is a constant reference for Jaspers as he develops his conception of world history.

Many of these references are negative, indicating Jaspers’s departures from Hegel. As I already indicated, Jaspers, pace Hegel, calls into question the continuing dominance of Europe, which he sees as being in crisis following the world wars. “The self-evident equation of a closed circle of Western culture with world history as such has been broken through,” he writes (Jaspers 1953, 69). Hegel’s assumption of lasting European dominance serves Jaspers as an indicator for some of the problems with his historical thought. In his estimation, Hegel had an insufficient grasp of how science and technology would develop as forces shaping history. These forces helped Europe extend its influence across the globe from about 1500 onward, but now they have turned against continuing European domination. Modern technological means of communication, in particular, have united the globe and dislodged Europe from its privileged position, Jaspers finds. More generally Jaspers seems to agree with critics like Friedrich August Hayek or Karl Popper that Hegel’s understanding of history is too monocausal, which is why his thought could become the basis for ideologies of total planning. Because it is not possible to know the totality of history with certainty, however, “[s]peculation on the future is precisely not insight into an unequivocal necessity, but into an open space of possibilities and probabilities” (Jaspers 1953, 188).

This critical stance vis-à-vis Hegel might suggest that Jaspers abandoned systematization altogether in *Origin and Goal*. He posits an “open space” of contingency against the closure of Hegel’s late work and its tendency to see an inexorable logic at play in the march of history toward its *telos* of freedom. However, as his comments in the introduction make clear, Jaspers’s allowances for contingency in history do not mean that he disavows systematization altogether. He does not hold a nominalist position, but rather believes that history is an indispensable guide on value questions because it has an origin and a goal, however elusive and unknowable these may be. Despite his numerous criticisms of Hegel’s position, he operates with similar assumptions. Thus, the actors on the stage of the historical drama as Jaspers tells it are civilizations as embodiments of spirit. The crucial change from Hegel, however, is that the great Eurasian civilizations do not appear, as in Hegel’s stadial conception of history, in succession, with one paving the way for the next as the site of Spirit coming into its own, but contemporaneously. The Indian and Chinese civilizations are on equal footing with Europe in the process of cultural development, not mere precursors.

Jaspers’s affirmation of unity in the diversity of cultural development hinges on the notion of the axial age. This period, which is common to the Eurasian civilizations, laid the spiritual basis for the major philosophical and religious traditions, thus giving rise to humans “as we know [them] today.” As such it is the source of a historical trajectory shared by the civilizations of Eurasia. At first this trajectory was a “relative universal,” shared only by the civilizations of Europe, the Middle East, and Asia. These civilizations were “islands of light amidst the broad mass of humanity” (Jaspers 1953, 23).^3^ Only later, as it was carried around the globe, this relative universal became an absolute universal. This universalization occurred during the period of European imperial expansion, which Jaspers calls the age of science and technology. Jaspers contends, much like he did in his debate with Karl Mannheim, that science and technology in themselves are without consequences for the realm of human values. In the words of Goedert (1998, 215), “[m]odern development based on the progress of science and technology does not bear on the universal-historic structure, which is essentially spiritual.” Such developments can only serve as a conduit for more consequential “spiritual” flows. The effect of European expansion during the modern era of science and technology, then, was not to spread a uniquely European spirit, but to universalize the values of the axial age (even while undermining them by hastening processes of rationalization). Thus, modernity is not exclusively a European project, but one that has deeper roots in the axial age. As such, it is a project which can be shared by any civilization.^4^

Jaspers identified an important problem of nineteenth-century world-historical accounts by questioning their self-evident equation of concepts like world history and modernity with the West. He seeks to overcome this aspect of universal-historical thinking by broadening the scope of his narrative. He remains in the paradigm of nineteenth-century universal history, however, insofar as his narrative is fundamentally about developments of “spirit” considered in isolation from other, more material factors. Jaspers draws a strong line of separation between the spiritual on the one hand, and science and technology on the other. This was already hard to defend when he formulated it in Heidelberg in the interwar years, as Mannheim’s critique makes clear. Today it must appear even more indefensible.

The Egyptologist and theorist of cultural memory Jan Assmann has recently suggested that “the decisive question is not so much what happened in the Axial Age but how these events have been remembered, represented, and reconstructed in cultural traditions” (Assmann 2012, 369).^5^ He suggests shifting the focus from the axial age as event—as a breakthrough that ushered in something historically new—to the axial age as myth that grounds traditions of reading and memorializing certain texts. Viewed in this light, the axial age cannot simply be understood as a cognitive advance that subsequently radiated outwards from the points of its inception, as Jaspers and many of the sociologists who have taken up his lead have done. Rather, we must view it in terms of mediations and material practices. The process whereby the axial as “relative universal” turned into the axial as “absolute universal,” then, must also be understood in terms of the interactions, encounters and circulations that made its spread around the planet possible. Here I suggest turning to a theorist who is seldom taken into consideration in these kinds of discussions. In fact, one consequence of the partial transplantation of the axial age discussion into U.S. academia in the course of the twentieth century has been that it started being debated in a Parsonsian framework, which famously excludes several important figures of sociology’s early decades, among them Du Bois. I hope to show that, by re-envisioning what texts and ideas should be part of the canon of sociology, we can begin to address some of the problems that beset contemporary social thought.

## World History and Double Consciousness

At his graduation from Fisk University in 1888, Du Bois gave an oration on Otto von Bismarck, the first chancellor of a unified German Empire who had been in office since 1871. Du Bois has often been viewed as a Germanophile since then. He loved Wagner operas but had curiously little to say about jazz and other African American forms of cultural expression (except for the “Sorrow Songs”). He not only studied in Germany in the early 1890s, maintained contact with German scholars (e.g., he met with Max Weber during his visit in the United States; see Scaff 2011), and returned there as late as 1936, after Hitler had already taken power (Sollors 1999). He was also drawn to aspects of German thought and drew on German ideas in his writings. This is the subject of a small but growing literature (Williamson 1984; Levering-Lewis 1995; Barkin 2000; Barkin 2005; Schäfer 2001; Edwards 2007; Kalbus 2009; Lenz 2012; see also Adell 1994, ch. 1). Still during Du Bois’s lifetime, Broderick (1958) noted that it was in Germany that Du Bois turned from a historian into a sociologist. In one of his autobiographical works, Du Bois writes of his studies in Berlin: “Under these teachers and in this social setting, I began to see the race problem in America, the problem of the peoples of Africa and Asia, and the political development of Europe as one” (Du Bois 1986a, 588).

Before his studies in Berlin, Du Bois spent four years at Harvard University doing both undergraduate and graduate work. There, Du Bois took a class with William James, who was at the time already a professor of philosophy. In his autobiographical writings written more than half a century later, Du Bois says that during that time he “became a devoted follower of James” (quoted in Zamir 1995, 11). It has been tempting for scholars to take this at face value and to read Du Bois’ early work through a pragmatist lens (for a recent example, see England and Keith Warner 2013), but as Zamir 1995 convincingly argues in his path-breaking book *Dark Voices*, the recollections that Du Bois wrote down late in life, at least in this regard, work as a red herring. He notes in particular that Du Bois’s early work does not align with James’s ahistorical conception of consciousness. While I cannot go into the details of Zamir’s argument on the divergence between James’s and Du Bois’s understandings of consciouness and the self here, I will note that my main take away from his work is the role Hegel had in animating the development of Du Bois’s original ideas on these issues at the intersection of philosophy, history and sociology.^6^

*Dark Voices* has been called “the first major attempt to reconstruct in detail Hegel’s role in Du Bois’ thought at this stage of his career” (Siemerling 2001, 327), referring to the time at the turn of the century when Du Bois wrote the essays that make up *The Souls of Black Folk*. In what follows, I will focus on this work by Du Bois, particularly its opening chapter, “Of Our Spiritual Strivings,” which was first published as an article in Atlantic Monthly in 1897, almost exactly a half century before the publication of Jaspers’s *Origin and Goal*.^7^ I will largely follow Zamir’s reconstruction of the role of Hegel’s thought in this work (see especially Zamir 1995, ch. 4).

Like Jaspers, Du Bois reads Hegel critically; Zamir notes that “Du Bois’s departures from the Hegelian narrative are crucial” (Zamir 1995, 114). First, it is important to note which Hegelian narrative Du Bois departs from. Unlike Jaspers, whose references are to the Hegel of the Berlin lectures on world history held in the 1820s, Du Bois’s main point of reference is Hegel’s earlier work, the *Phenomenology of Spirit *(1807), particularly its middle section. That is not to say that Du Bois was unaware of Hegel’s later work on the philosophy of history, or that he ignored it altogether, but the emphasis is clearly on the younger Hegel. By focusing on the middle section of the *Phenomenology* which deals with the figure of the “unhappy consciousness,” Du Bois preserves the tension in Hegel’s narrative of spirit’s progressive unfolding, and does not arrive at a resolution in the shape of absolute spirit.

Hegel wrote the *Phenomenology* in the midst of the Napoleonic wars, and he was concerned with the implications of the wars’ outcome for historical time-much like Du Bois and Jaspers were with respect to the wars that had recently ended when they wrote the works discussed in this paper. Would the victory of Napoleon’s troops, which he desired, lead to cultural and political renewal and bring the German states out of the historical backwater they appeared consigned to? György Lukács writes in his study *The Young Hegel*,

This connection between time and philosophy is the lasting foundation of Hegel’s conception of the history of human thought. For that very reason it is vital to realize that when he wrote the *Phenomenology* he conceived it as the intellectual form of a newly-born configuration of world history, whereas, as we shall see, his view of the relation of his philosophy to world history undergoes a radical change later on, even though he does not deviate from the same general principles. (Lukács 1975, 454).

Whereas the young Hegel sees himself in the role of midwife to new cultural and political forms engaging in an ongoing struggle over the shape of the world, the later Hegel’s work has undergone what Lukács calls a “‘reconciliation’ with reality.” He no longer sees himself as writing philosophy for the world to come. His famous dictum in the *Elements of the Philosophy of Right* that “the Owl of Minerva spreads its wings only with the falling of the dusk” (Hegel 1955, 17) points to this reorientation. His task now is to account for how the world came to be the way it is, not to aid in the birthing of a new one. By the time he writes the *Philosophy of Right*, Hegel “gives an entirely opposed picture of the relation between his philosophy and the present” (Lukács 1975, 456). Thus, in the later Hegel, there is a tendency toward forming closed systems in which history is no longer unfolding, but in which major conflicts and contradictions have by and large found their rigidified resolution. In other words, between the “logical” and the “historical” dimensions of Hegel’s dialectic, the logical ultimately wins the upper hand in his late work. While in the story of the *Phenomenology* the “absolute idea” remains a receding horizon, the reconciliation of contradictions is an attained endpoint in the Berlin lectures. Hegel’s later lectures famously relegate those who have not attained statehood to historical insignificance (Hegel 1997), but in the *Phenomenology* the subjugated are still involved in a struggle for world-historical significance (understood in idealist terms as a struggle for recognition).

This is where Du Bois picks up, and in this he anticipates the accounts of Wahl (1951) and Hyppolite (1974), among others, who see the unhappy consciousness as the central theme of the *Phenomenology* and maintain that the work is not teleological, as is commonly argued by Hegel critics. As Zamir puts it, “Du Bois’s emphasis is not on the singular *Geist* but on soul*s*” (Zamir 1995, 115). This was a remarkable move, because, as Zamir points out, most American philosophers at the time used Hegel as “a prophet of American exceptionalism” providing “metaphysical alibis for manifest destiny” (Zamir 1995, 117, 126). Thus John Dewey, for instance, “always subsumes his particularist understandings into higher syntheses” (Zamir 1995, 121). Josiah Royce, whom Du Bois encountered while a student at Harvard University, also characterized Hegel’s thought in idealist terms as being about the reconciliation of contradictions. In his efforts to stave off systemic closure, Du Bois also seems to anticipate Jaspers. However, Du Bois indicates a different path in which such a world-historical account can be developed.

Du Bois makes use of Hegel in elaborating his famous concept of “double-consciousness,” which he develops on the basis of his own experiences.

I remember when the shadow swept across me. I was a little thing, away up in the hills of New England, where the dark Housatonic winds between Hoosac and Taghkanic to the sea. In a wee wooden schoolhouse, something put it into the boys’ and girls’ heads to buy gorgeous visiting-cards—ten cents a package—and exchange. The exchange was merry, till one girl, a tall newcomer, refused my card,-refused it peremptorily, with a glance. Then it dawned upon me with a certain suddenness that I was different from the others; or like, mayhap, in heart and life and longing, but shut out from their world by a vast veil. (Du Bois 2007, 8).

This episode is Du Bois’s retelling of the famous passage in the *Phenomenology* on “Lordship and Bondage,” often called the “master-slave dialectic.” In the *Phenomenology* this section describes a stage in the development of self-consciousness when self-consciousness attains greater realization of self through confrontation with another. In Hegel’s narrative, this confrontation takes the form of a life-and-death struggle (Hegel 1977). In Du Bois’s story from the wee wooden schoolhouse, the encounter with the other does not come to a head in this way, and yet it contains many of the elements of Hegel’s dialectic of recognition. In fact, Zamir points out that in Du Bois’s story, two moments that are distinct in the Hegelian account—the move from consciousness to self-consciousness and the confrontation of one self-consciousness with another—are collapsed into one, indicating that, in Du Bois’s understanding, the development of consciousness for black Americans is always relational and thus political. Furthermore, where in Hegel the curtain of mere appearances is drawn away with the emergence of consciousness (Hegel 1977, 103), in Du Bois the veil descends in the moment of confrontation with the other in the shape of the “tall newcomer.” This division in his consciousness—and the consciousness of black Americans more generally^8^—outlasts the moment of the peremptory glance. At first, it induces a withdrawal from the world around him:

I had thereafter no desire to tear down that veil, to creep through; I held all beyond it in common contempt, and lived above it in a region of blue sky and great wandering shadows. The sky was bluest when I could beat my mates at examination-time, or beat them at a foot-race, or even beat their stringy heads. (Du Bois 2007, 8).

The “region of blue sky” in which Du Bois dwells is the false sense of being able to transcend the negative experience of the encounter with the other without having to work through it. This reaction corresponds with the moment of skepticism in Hegel’s account, which he likens at one point (using an image very similar to Du Bois’s stories about his schoolmates) to “the squabbling of self-willed children, one of whom says *A* when the other says *B*, and in turn says *B* when the other says *A*, and who by contradicting themselves buy for themselves the pleasure of continually contradicting one another” (Hegel 1977, 126). This pleasure does not last long, however; eventually the blue sky begins to fade and Du Bois’s protagonist must come to terms with the material reality of unfreedom. This coming-to-terms is no simple reconciliation with the way things are, but results in what Hegel describes as “the consciousness of self as a dual-natured, merely contradictory being” (Hegel 1977, 126).

This process affecting the self also affects the group, and when, in the following paragraph, Du Bois makes the shift to the collective level, the implications for the understanding of world history become clearer:

After the Egyptian and Indian, the Greek and Roman, the Teuton and Mongolian, the Negro is a sort of seventh son, born with a veil, and gifted with second-sight in this American world,-a world which yields him no true self-consciousness, but only lets him see himself through the revelation of the other world. It is a peculiar sensation, this double-consciousness, this sense of always looking at one’s self through the eyes of others, of measuring one’s soul by the tape of a world that looks on in amused contempt and pity. One ever feels this two-ness,—an American, a Negro; two souls, two thoughts, two unreconciled strivings; two warring ideals in one dark body, whose dogged strength alone keeps it from being torn asunder. (Du Bois 2007, 8).

The six civilizations listed before “the Negro” do not correspond exactly to the so-called axial civilizations of Eurasia, but they are civilizations generally regarded as having had world-historical significance. Alongside these, other groups bear the stigma of the laggard. But this status as “seventh son” is no mere handicap; second-sight also gives what might be called (following feminist epistemology) epistemic advantage, or insight into the ambivalences and undersides of the cultural present (what Du Bois elsewhere calls “afterthought”). In the words of Paul Gilroy, “cultures of diaspora blacks can be profitably interpreted as expressions of and commentaries upon ambivalences generated by modernity and their locations within it” (Gilroy 1993, 117). Thus, although black Americans have been exposed to what Jaspers calls the axial “spiritual radiation” (Jaspers 1953, 7) of Eurasian origin and may aspire to become, in Du Bois’s words, “a co-worker in the kingdom of culture” (Du Bois 2007, 9), their location within this cultural formation is marked by lasting ambivalence.

This sense of enduring ambivalence recurs in several of Du Bois’ later writings. In “The Development of a People,” he discusses the relation between cultural development and social processes “in a world which is daily becoming physically smaller” (Du Bois 1904, 292). He highlights the role of “culture-contact” in the development of cultural groups once they have satisfied basic material needs: “as the group meets other groups and comes into larger spiritual contact with nations, there is that transference and sifting and accumulation of the elements of human culture which makes for wider civilization and higher development” (Du Bois 1904, 295). Du Bois uses his detailed knowledge of the political economy of the slave trade (the subject of his dissertation) to demonstrate how this momentous historical event had a “crippling” effect on this process of cultural development for black Americans, as a result of which they are not recognized as full participants in “wider civilization.” Thus, although the world is shrinking and enabling greater contact between civilizations, the veil of the color-line continues to make full recognition impossible.

After the First World War, Du Bois wrote an essay whose title, “The Souls of White Folk,” refers back to his famous collection. Here he traces Europe’s (or white Euro-America’s) changing understanding of history. Until early modernity, “we were hammering our national manikins into one, great, Universal Man” (Du Bois 1986b, 923), but with the modern invention of whiteness, Euro-America was able to redefine human history simply as white history. This enabled Euro-America to hold up civilizational values while at the same time ignoring and violating them around the world with impunity. The Great War is just the latest in a line of disastrous results of this cultural hubris. What Du Bois describes can be understood as the counterpart to the veil, the unwillingness and inablity of the master to recognize his dependence on the subjugated.

To conclude this section, consider this passage from Du Bois’ 1940 autobiographical work, *Dawn of Dusk*:

In the folds of this European civilization I was born and shall die, imprisoned, conditioned, depressed, exalted and inspired. Integrally a part of it and yet, much more significant, one of its rejected parts; one who expressed in life and action and made vocal to many, a single whirlpool of social entanglement and inner psychological paradox, which always seem to me more significant for the meaning of the world today than other similar or related problems. (Du Bois 1986a, 555).

Modernity to Du Bois is not a shared project, but rather an experience premised on the divisiveness of the color-line.

## Conclusion

What have we learned by reviewing these two theorists’ departures from nineteenth-century thinking about big questions? Although both were influenced by the philosophy of Hegel, their work is informed by different parts of his oeuvre.^9^ Jaspers is influenced by the late Hegel’s conception of world history as a stadial succession of civilizations on the world stage. Though he takes issue with the notion that different cultures represent different stages of cultural development, he adopts the premise that cultural history is a story about civilizations as bounded entities. The civilizations associated with the axial age simultaneously and independently arrived at a cultural breakthrough that later spread to civilizations that did not take part in the initial breakthrough, including Africa and the Americas. Du Bois, in contrast, drew on the middle sections of the *Phenomenology of Spirit*. Rather than reading the relational process Hegel describes here as a mere intermediate step in the progressive unfolding of spirit en route to greater self-consciousness, Du Bois draws on Hegel’s figure of the “unhappy consciousness” to give due consideration to the lasting ambivalences and misrecognitions that beset cultural development. His account of cultural processes suggests that culture does not spread immediately through what Jaspers called “spiritual radiation,” or what subsequent theorists in sociology have described as an evolutionary process leading to adaptation to a new cognitive pattern. Rather, the spread of cultural patterns involves encounters and social interactions. In short, Du Bois suggests, it is an inherently relational process.

His approach suggests that the spread of the axial age to cultures not originally associated with the axial age meant there were encounters between cultures that often took the form of Hegel’s life-and-death struggle. The creation of the Atlantic world at the onset of what Jaspers called the age of science and technology was the beginning of many such confrontations between figurative and literal masters and slaves, both in the course of the slave trade and in the settler societies on its receiving end. Du Bois’s account of double-consciousness renders the notion that such encounters between the axial and the nonaxial world simply spread already existing values with deep roots in the Eurasian landmass very questionable. His work reminds us that to think about big questions of cultural and social change today, it is important to unpack abstractions and understand processes in terms of social interactions. Only in this way can we account for what Zamir (1995, 115) calls “the negativity of historical experience.”

W. E. B. Du Bois’ works highlights both the analytical and normative failings that result from freezing, by way of what Elias 1998 called “process-reduction,” civilizing processes into static civilizations. Although Du Bois is not usually counted among the founding figures of what in American sociology has become known as “relational sociology” (Emirbayer 1997),^10^ I believe that the discussion of his work in this paper suggests that we would do well to consider him as a relational theorist of cultural processes. As such, he can serve as a valuable guide to the study of big questions today.

## Footnotes

1 Scholars have identified similar themes in the works of Georg Simmel (Franke 1972; Levine 2004) and John Stewart-Glennie (Halton 2014), among others. Lewis Mumford’s *Transformations of Man* (Mumford 1956), which grew out of his work on the culture of cities and was conceived as a “pocket-sized alternative” to Toynbee (Miller 1989: 455), posits a more expansive period of “axial man” lasting for over 1,200 years, from 600 BCE to 650 CE. His discussion of this period has some similarities with Jaspers’ account of the axial age in that it, too, focuses on the rise of religions with a universal thrust and expanding communications across large territories. Despite these similarities, however, and partly due to the difference in periodization, Mumford rarely forms part of the axial age debate.

2 “On the Spirit of Europe” (*Vom europäischen Geist*) was the name of Jaspers’s presentation to the first Rencontres Internationales in Geneva in 1946; see Boy and Torpey 2013.

3 For a critique of Jaspers’s “central perspective” on world history, see A. Assmann 1989.

4 The discussion of “multiple modernities” is based in part on this aspect of the axial age thesis; see Eisenstadt 2000.

5 Jan Assmann has recently come under fire for the alleged anti-Semitic implications of his writings on the origins of monotheism. Here I will not address this controversy but rather clarify that I am turning to Assmann’s work for its conceptual contributions to the axial age debate, not for its account of the history of ancient Judaism, the “Mosaic distinction,” or monotheism more genearlly.

6 It is likely that Du Bois read Hegel in the German original while studying with George Santayana at Harvard University (see Zamir 1995; Weatherford-Jacobs 2002).

7 An English-language summary of the argument of *Origin and Goal* was published in the November 1948 issue of the New York-based magazine Commentary at the invitation of Hannah Arendt.

8 Though (Zamir 1995, 115) points out that the notion of double-consciousness is a class-specific diagnosis: it is not a characterization of all black Americans, but of the post-Reconstruction black middle class.

9 For an interesting perspective insisting there are several Hegels, see Malabou 1996.

10 In fact, the champions of this approach tend to reference theorists that were shaped by American pragmatism, such as George Herbert Mead.
